# Microbial optimization for improved charring of agricultural solid waste: a cutting-edge technology across the life cycle

**DOI:** 10.3389/fmicb.2024.1521639

**Published:** 2024-12-23

**Authors:** Yang Feng, Nazhafati Muhanmaitijiang, Jianqing Ye, Haoming Chen, Xiaolin Jia

**Affiliations:** ^1^School of Art and Design, Xijing University, Xi'an, China; ^2^School of Environmental and Biological Engineering, Nanjing University of Science and Technology, Nanjing, China; ^3^School of Art, Xi'an University of Science and Technology, Xi'an, China

**Keywords:** microbial modification, biochar, carbon sequestration, energy efficiency, agri-environment

## 1 Introduction

The resource utilization of agricultural solid waste plays a crucial role in environmental pollution prevention and agricultural sustainable development. Pyrolysis technology, which involves the thermal decomposition of organic materials in the absence of oxygen, offers characteristics such as convenient operation, carbon sequestration and emission reduction, as well as environmental friendliness. These features have made it gradually become one of the efficient ways for the resource utilization of agricultural solid waste. Under anaerobic (or oxygen-free) conditions, pyrolysis technology can effectively convert agricultural waste into valuable resources such as biochar, syngas, and bio-oil.

Notably, biochar, a product of the carbonization process during pyrolysis, demonstrates significant potential for application in the realms of wastewater treatment and soil enhancement (Khedulkar et al., 2023). The pyrolysis technology is particularly significant as it enhances the treatment efficiency of agricultural waste and fosters the advancement of a circular agricultural economy (Ferrari et al., 2024). However, the pyrolysis carbonization conditions have a crucial impact on the yield and quality of the carbonization products (Khedulkar et al., 2023). Currently, technologies for improving the quality of biochar from agricultural solid waste mainly focus on three aspects: raw material improvement, carbonization process optimization, and product modification. Despite extensive engineering studies on pyrolysis carbonization processes, practical applications still encounter challenges, including high energy consumption, low efficiency, and the emission of harmful gases (Sun et al., 2022; Lan et al., 2024). Therefore, the optimization of raw materials and products is the most critical improvement measure. An effective and environmentally friendly optimization technology is essential for enhancing the carbonization efficiency of agricultural solid waste, improving the quality of carbonization products, and reducing environmental impacts.

## 2 Microbial optimization technology represents a cutting-edge approach within the charring process of agricultural solid waste

In order to meet the challenge of upgrading the charring process, microbial optimization technology (MOT) has emerged as a promising approach in the charring process of agricultural solid waste. This technique primarily employs biological methods, including microbial degradation for raw material pretreatment and microbial colonization for product optimization, to enhance the treatment of biochar raw materials and improve the properties of the final products (Pham et al., 2023; Peng et al., 2024). This technique eliminates the need for chemical reagents or large-scale physical modification equipment, yet significantly enhances multiple properties of biochar, including improved pollutant degradation efficiency, enriched soil nutrients, and optimized soil microbial community structure (Lan et al., 2024). Furthermore, the microbial modification process is gentle and energy-efficient, aligning perfectly with the sustainable development concept of green and low-carbon emissions (Dong et al., 2024). Numerous microorganisms possess unique physiological functions. For example, fungi such as white-rot fungi, brown-rot fungi can efficiently degrade lignocellulose (Gao et al., 2016), promoting the formation of a honeycomb-like three-dimensional loose structure in biomass, which lays a solid foundation for subsequent efficient carbonization processes (Wang et al., 2018). Bacteria with phosphate-solubilizing functions, such as *Enterobacter* and *Bacillus*, efficiently convert insoluble phosphorus into forms that can be directly utilized by organisms through the secretion of organic acids and extracellular enzymes (Chen et al., 2023). This not only significantly improves the nutrient utilization rate, surface functional group activity, and anion content of biochar, but also aids in the immobilization of free heavy metal cations in the environment, thereby achieving multiple environmental benefits (Peng et al., 2024; Chen et al., 2019).

## 3 MOT can significantly increase the efficiency and reduce the environmental impact of the carbonization process

Microorganisms play a crucial role in both the pretreatment of raw materials and product optimization in biochar production, offering environmentally friendly solutions to the challenges faced by this process. During the raw material pretreatment stage, MOT primarily utilizes enzymes secreted by microorganisms to efficiently decompose the complex lignocellulosic structures in biomass (Gao et al., 2016; Pham et al., 2023). Studies have shown that microbial pretreatment significantly affects the initial temperature, maximum weight loss rate, and activation energy during the biomass pyrolysis process (Sun et al., 2022). For example, pretreatment with brown-rot fungi can reduce the activation energy during the biomass (*Pinus massoniana*) pyrolysis and carbonization process by 42.3 kJ/mol and lower the initial pyrolysis temperature by 11°C. This substantially reduces the energy consumption of the pyrolysis process (Gao et al., 2016). In the product optimization stage, microorganisms mainly colonize the surface of biochar, improving its pore structure, specific surface area, and surface functional groups through metabolic activities and secretions, thereby enhancing its functionality (Peng et al., 2024). For instance, colonization by *Aspergillus niger* can increase the specific surface area of biochar by 815.03% and significantly increase the number of functional groups such as amides, alkanes, carboxyl groups, and hydroxyl groups. Thus, it results in a 61% improvement in the removal efficiency of atrazine by biochar (Yu et al., 2020). Compared with traditional physical and chemical methods, MOT reduces the use of chemical reagents and utilizes a more gentle preparation process (Dong et al., 2024). Therefore, the application of MOT in the carbonization process not only improves the pyrolysis efficiency of biomass and the performance of biochar but also significantly reduces the negative impact on the environment by reducing the use of chemical reagents and lowering energy consumption.

## 4 Enhancement of agricultural organic solid waste carbonization process by MOT from a full lifecycle perspective

**Figure 1:**
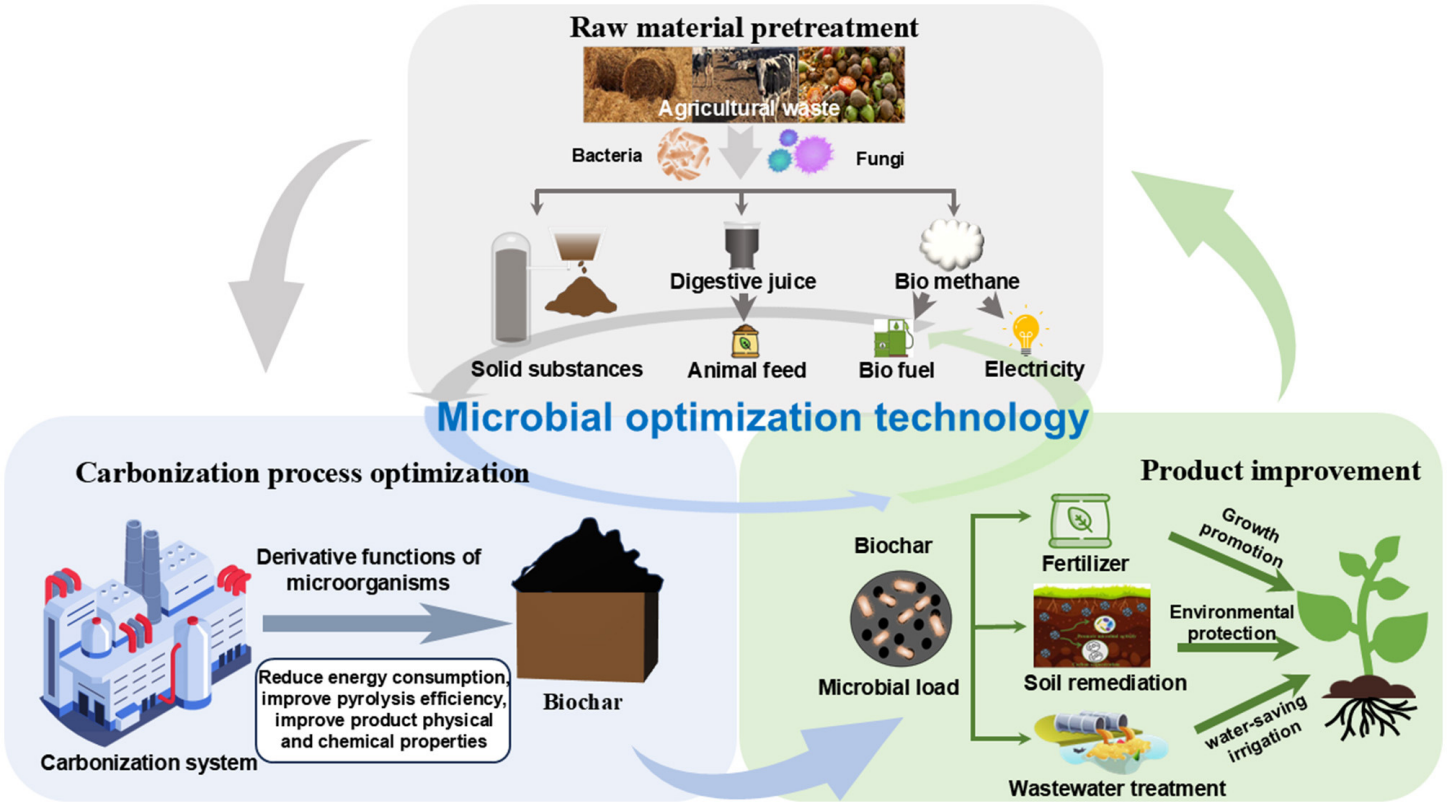
Application of functional microbial optimization technology to the full life cycle of an agricultural organic solid waste carbonization process.

Throughout the entire lifecycle of the carbonization process, from raw material pretreatment to the final production of biochar, MOT exhibits positive effects (Figure 1). Firstly, during the raw material pretreatment stage, MOT converts agricultural organic solid waste into value-added products, significantly enhancing resource recycling efficiency. This reduction in dependence on fossil resources also effectively alleviates environmental pollution issues (Khaswal et al., 2024). Microbial pretreatment of agricultural solid waste yields biogas, digestate, and solid residues, all of which can be further utilized as resources with relatively low energy consumption (e.g., for power generation, fertilizer production, and feed) (Awogbemi and Kallon, 2022; Haque et al., 2023; Fuentes-Grünewald et al., 2021). Secondly, during the carbonization and pyrolysis process, microbially modified agricultural organic solid waste is more prone to forming abundant pore structures, which contributes to improve pyrolysis efficiency and reduce energy consumption (Ferrari et al., 2024). Lastly, the biochar produced after pyrolysis, when further modified by microorganisms, can serve as a soil conditioner, significantly enhancing soil fertility and promoting plant growth (Schommer et al., 2024). Overall, MOT achieves environmental protection and low-carbon goals by reducing energy consumption and environmental pollution across the entire lifecycle of the carbonization process. The development of this technology provides strong technical support for the green and sustainable development of agricultural organic solid waste carbonization processes and the improvement of agricultural production quality.

## 5 Feasibility and future prospects of MOT application in carbonization of agricultural organic solid waste

The application of MOT provides an environmentally friendly and sustainable method for improving the efficiency of carbonization processes and expanding the application potential of carbonized products. In practical engineering applications of this technology, we need to focus on the following core issues: Firstly, it is essential to explore comprehensive application strategies for MOT in the carbonization process. This involves identifying and selecting the most suitable MOT application methods to meet different process needs, ultimately achieving energy conservation, emission reduction, and environmental pollution mitigation. Secondly, future research should focus on optimizing microbial strains to ensure their adaptability to diverse biomass materials and carbonization conditions, thereby enhancing the overall efficiency of the carbonization process and improving the functionality of biochar. Thirdly, environmental risk assessments must be strengthened to ensure that microbial optimization measures do not pose any potential negative impacts on the environment and human health. Finally, emphasis should be placed on innovation and application, actively pursuing the integration of MOT with other advanced technologies (e.g., electrochemical advanced oxidation, metal catalyst composite preparation, strain genetic enhancement, etc.), fostering international cooperation, and conducting interdisciplinary research, all of which are crucial for achieving large-scale MOT application and global environmental sustainability.
